# MRI-assessed atrophy subtypes in Alzheimer’s disease and the cognitive reserve hypothesis

**DOI:** 10.1371/journal.pone.0186595

**Published:** 2017-10-16

**Authors:** Karin Persson, Rannveig Sakshaug Eldholm, Maria Lage Barca, Lena Cavallin, Daniel Ferreira, Anne-Brita Knapskog, Geir Selbæk, Anne Brækhus, Ingvild Saltvedt, Eric Westman, Knut Engedal

**Affiliations:** 1 Norwegian National Advisory Unit on Ageing and Health, Vestfold Hospital Trust, Tønsberg, Norway; 2 Department of Geriatric medicine, Oslo University Hospital, Ullevaal, Nydalen, Oslo, Norway; 3 Department of Geriatric Medicine, The memory clinic, Oslo University Hospital, Ullevaal, Nydalen, Oslo, Norway; 4 Department of Neuromedicine and Movement Science, Norwegian University of Science and Technology (NTNU), Trondheim, Norway; 5 Department of Clinical Science, Intervention, and Technology, Division of Medical Imaging and Technology, Karolinska Institute, Stockholm, Sweden; 6 Department of Radiology, Karolinska University Hospital, Stockholm, Sweden; 7 Division of Clinical Geriatrics, Department of Neurobiology Care Sciences and Society, Karolinska Institutet, Stockholm, Sweden; 8 Centre for Old Age Psychiatric Research, Innlandet Hospital Trust, Ottestad, Norway; 9 Institute of Health and Society, University of Oslo, Oslo, Norway; 10 Department of Neurology, Oslo University Hospital, Ullevaal, Nydalen, Oslo, Norway; 11 Department of Geriatrics, St Olav Hospital, University Hospital of Trondheim, Trondheim, Norway; Nathan S Kline Institute, UNITED STATES

## Abstract

**Background/Aims:**

MRI assessment of the brain has demonstrated four different patterns of atrophy in patients with Alzheimer’s disease dementia (AD): typical AD, limbic-predominant AD, hippocampal-sparing AD, and a subtype with minimal atrophy, previously referred to as no-atrophy AD. The aim of the present study was to identify and describe the differences between these four AD subtypes in a longitudinal memory-clinic study.

**Methods:**

The medial temporal lobes, the frontal regions, and the posterior regions were assessed with MRI visual rating scales to categorize 123 patients with mild AD according to ICD-10 and NINCDS-ADRDA criteria and the clinical dementia rating scale (CDR) into atrophy subtypes. Demographic data, neuropsychological measures, cerebrospinal-fluid biomarkers, and progression rate of dementia at two-year follow-up were compared between the groups.

**Results:**

Typical AD was found in 59 patients (48%); 29 (24%) patients had limbic-predominant AD; 19 (15%) had hippocampal-sparing AD; and 16 (13%) belonged to the group with minimal atrophy. No differences were found regarding cognitive test results or progression rates between the different subtypes. Using adjusted logistic regression analysis, we found that the patients in the minimal-atrophy group were less educated, had a lower baseline CDR sum of boxes score, and had higher levels of amyloid β in the cerebrospinal fluid.

**Conclusion:**

Previous results concerning the prevalence and the similar phenotypic expressions of the four AD subtypes were confirmed. The main finding was that patients with minimal atrophy as assessed by MRI had less education than the other AD subtypes and that this could support the cognitive reserve hypothesis and, at least in part, explain the lower degree of atrophy in this group. Patients with less formal education might present with clinically typical AD symptoms before they have positive biomarkers of AD and this finding might challenge suggested biomarker-based criteria for AD.

## Introduction

The typical symptomatology of Alzheimer’s disease (AD) includes the impairment of episodic memory [1], but atypical presentations with other debut symptoms such as language, visuospatial, or behavior predominant dysfunction have been found to exist in approximately 6% to 30% of AD patients [2, 3]. Similarly, the typical pathological pattern of AD includes the accumulation of amyloid plaques and neurofibrillary tangles distributed in a characteristic way [4, 5], but in approximately one of four patients, the typical medial temporal lobe and associative cortex distribution of neurofibrillary tangles is lacking [6]. Instead, limbic-predominant or hippocampal-sparing atypical presentations have been identified [6].

It has been demonstrated that volumetric MRI measurements of regional brain atrophy correlate with the distribution and degree of neurofibrillary tangle pathology [7, 8], making MRI a potential surrogate marker of regional tangle distribution. Furthermore, a correlation between the previously described neuropathologically defined subtypes and volumetric MRI has been confirmed [9].

Several recent studies have used MRI measures as in vivo markers to explore the aforementioned tangle/atrophy-based AD subtypes [10–12]. Byun et al. used voxel-based morphometry (VBM) and Ferreira et al. used visually assessed MRI to categorize highly selected, longitudinally assessed patients from the ADNI cohort into subgroups [10, 11, 13]. These studies identified the same three AD subtypes that had been found in previous neuropathological studies: typical AD, limbic-predominant AD, and hippocampal-sparing AD. In addition, a fourth group lacking evident atrophy in both medial temporal and associative cortex areas, previously called no-atrophy AD, was reported. As it is difficult to completely rule out some degree of atrophy, a more suitable term for this entity may be minimal-atrophy AD, and this term is used for this subtype in the current study. This subtype attracts special interest, as one of the major hallmarks of AD, i.e., atrophy, is lacking or at least minimal, thereby questioning the diagnostic value of MRI-evaluated atrophy in AD, especially as a mandatory diagnostic criterion.

Thus, the aim of the present study was to explore whether we could find the same four subtypes in a sample of memory-clinic patients who have been followed up for two years, as have previously been found in highly selected ADNI patients. Secondly, we wanted to describe differences between the subtypes with regard to demographic data, neuropsychological measurements, neuropsychiatric symptomatology, and cerebrospinal fluid biomarkers, with a special emphasis on the minimal-atrophy group. Thirdly, we wanted to examine the progression rate of the subtypes, as this has not been done in a clinical, naturalistic AD cohort before.

## Materials and methods

### Participants

The patients were recruited from a larger study for which the inclusion process has been described thoroughly in a previous paper [14]. For the present study, only patients with a baseline diagnosis of AD with a mild degree of dementia were included (n = 155). Of these, 123 patients had been assessed with MRI of the brain within six months prior to or after the clinical examination at baseline (in 91% of the patients, less than four months between clinical examination and MRI) and had been followed up after 24 months on average (range 17–34 months).

### Diagnoses

Two of the authors (MLB and KP) had conducted an interrater reliability analysis of 51 patients including eight different diagnoses, demonstrating very good interrater agreement for early- and late-onset AD (kappa 0.73 and 0.85 respectively) [14, 15]. Therefore, patients with AD were diagnosed by one of these authors alone or by two other authors in consensus (RSE and IS), according to the ICD-10 and NINCDS-ADRDA criteria based on all available clinical information, without knowledge of the results of the MRI visual rating scales. Patients with mixed AD/vascular disease (n = 3) were regarded as having AD. All patients had mild dementia according to the ICD-10 and CDR score of 0.5 or 1.0. Although clinical MRI reports and the results of the CSF biomarkers were available, this information was included only in the diagnostic workup according to the diagnostic criteria [16, 17]. APOE-ɛ4 status was not available during the diagnostic workup.

At follow-up, the patients were diagnosed again by the same authors. All patients except for two retained their baseline diagnosis (MCI due to AD at follow-up, one with limbic-predominant and one with typical AD pattern).

### Clinical assessment

All patients were clinically assessed with a standardized examination protocol [18]. For the present study, the following cognitive measures were used to characterize the patients: the Mini-Mental State Examination–Norwegian Revised Version (MMSE-NR), a measure of global cognitive function that can be scored from zero to 30, with a higher score denoting better cognitive functioning [19, 20]; the clock-drawing test, measuring visuoconstructional and executive functions on a scale from zero to 5, with higher scores indicating better functioning [21]; the Trail Making Test A and B to assess psychomotor speed and executive function, measured by the number of seconds used to complete the test, with a longer time indicating more impaired functioning [22] (the result was dichotomized to 1 if the patient performed the test better than 180/360 seconds (test A/test B), and 0 for results above 180/360 seconds, or if the patient was too cognitively impaired to perform the test); the Consortium to Establish a Registry for Alzheimer’s Disease (CERAD) 10-word delayed recall test with scores from zero to 10, with a higher score denoting better delayed recall function [23]; figure copying from the CERAD constructional praxis exercise to assess visuospatial skills on a scale from zero to 11, with higher scores indicating better functioning [23]; a 15-word short version of the Boston Naming Test (BNT) [24] to assess word retrieval; the animal-naming test to assess semantic fluency [25]; and the controlled oral word association test (COWAT-FAS test) to assess phonemic fluency [26].

The Clinical Dementia Rating scale (CDR) was used as a global measure of dementia. The scale includes six items that assess cognition and activities of daily living (memory, orientation, judgment and problem solving, community affairs, home and hobbies, and personal care). Each item on the CDR can be scored as zero, 0.5, 1, 2, or 3; the higher the score, the more severe the impairment. The subscores can be added, yielding a sum score between zero and 18 points—the CDR Sum of Boxes (CDR-SB) [27]. In addition to the sum score, a total CDR score can be calculated using an algorithm that gives priority to the memory item, reflecting the stage of dementia: 0 (no dementia), 0.5 (questionable dementia), 1 (mild dementia), 2 (moderate dementia), or 3 (severe dementia). The CDR was scored by the same CDR-certified co-authors (KP, RSE, or MLB) at baseline and follow-up, based on all available clinical data. The raters were blinded to the baseline CDR score when assessing CDR at follow-up and vice versa. The progression rate was calculated as the annual change in CDR-SB.

To assess depressive symptoms during the previous week, the Cornell Scale for Depression in Dementia (CSDD), comprising 19 items with possible scores from zero to 2, was administered. The maximum sum score is 38 points; the higher the score, the more severe the depressive symptoms are [28]. To assess neuropsychiatric symptoms, the Neuropsychiatric Inventory Questionnaire (NPI-Q), a 12-item informant scale, was administered [29]. NPI-Q rates the severity of neuropsychiatric symptoms on a scale from zero to 3, for a total score of zero to 36; the higher the score, the more severe the neuropsychiatric symptoms are.

Data on previous cerebrovascular disease and risk factors were registered from the patients’ records.

### Magnetic resonance imaging

The MRI examinations had been conducted at different locations with somewhat different protocols but all with coronal, transverse and sagittal imaging, in 63 of the cases with 3DT1 and T2 sequence in the other 60 patients. Field strength (1.5 T or 3 T) also differed. The distribution of scans with or without 3DT1 and 1.5 or 3 T did not differ between the subtypes (Table 1). A neuroradiologist (LC) with extensive experience evaluating cerebral atrophy using rating scales examined the MRI scans blinded to any clinical data [30, 31]. Medial temporal lobe atrophy (MTA) was assessed in both hemispheres using the Scheltens scale, including evaluation of the choroidal fissure, the temporal horn of the lateral ventricles, and the height of the hippocampus on a scale from zero to four [32]; the higher the score, the more atrophy is present. A mean of the two hemisphere scores was calculated. Frontal atrophy was assessed using the global cortical atrophy frontal subscale (GCA-f) [33, 34], evaluating the width of the sulci and the volume of the gyri in the frontal lobes on a scale from zero to three; the higher the score, the greater the amount of atrophy. Posterior atrophy was assessed using the Koedam scale (PA), including evaluation of the posterior cingulate, the parieto-occipital sulcus, the precuneus, and the widening of sulci and volume of gyri in the parietal lobes, on a scale from zero to three; the higher the score, the greater the atrophy [35]. The neuroradiologist has demonstrated excellent intrarater agreement for the MTA scale and substantial agreement for the other two scales in previous studies [30, 31].

**Table 1:** Baseline characteristics of the patients by AD subtype based on age-adjusted visual rating scale measures of the atrophy in the medial temporal lobes (Scheltens MTA scale), frontal lobes (GCA-f) and posterior regions (Koedams scale).

	Minimal-atrophy n = 16, 13.0%	Limbic-predominant n = 29, 23.6%	Hippocampal-sparing n = 19, 15.4%	Typical AD n = 59, 48.0%	p*	p**
Age, years (SD)	73.4 (7.5)	75.1 (6.2)	71.4 (9.9)	74.3 (6.7)	0.620	0.735
Female gender, %	68.8%	58.6%	47.4%	54.2%	0.298	0.274
Education, years (SD; range)	9.4 (3.1; 7–18)	11.4 (3.1; 7–18)	11.7 (4.2; 7–20)	11.9 (3.6; 7–20)	**0.013**	**0.014**
0–7 years, n (%)	7 (43.8)	4 (13.8)	3 (15.8)	5 (8.5)	**0.001**	**0.001**
8–13 years, n (%)	7 (43.8)	19 (65.5)	10 (52.6)	36 (61.0)	0.216	0.198
>13 years, n (%)	2 (12.5)	6 (20.7)	6 (31.6)	18 (30.5)	0.149	0.186
Symptom duration, years (SD)	2.7 (2.3)	2.9 (2.0)	3.9 (2.7)	3.2 (2.4)	0.494	0.417
Age at onset^a^	70.7 (8.3)	72.2 (6.8)	68.4 (11.1)	71.4 (7.2)	0.741	0.844
Mean follow-up time, months (SD)	22.7 (2.8)	23.0 (3.4)	25.0 (3.5	23.5 (2.7)	0.306	0.265
MMSE	20.7 (4.6)	22.1 (4.7)	22.8 (4.8)	22.3 (3.9)	0.170	0.157
Clock drawing test	3.6 (1.5)	3.5 (1.6)	3.0 (1.6)	3.1 (1.7)	0.298	0.378
TMT A below 180 sec, n (%)	16 (100)	19 (73.1)	17 (89.5)	47 (82.5)	0.071	0.059
TMT B below 360 sec, n (%)	3 (23.1)	12 (57.1)	6 (33.3)	25 (45.5)	0.140	0.122
Delayed recall	1.6 (1.8)	1.6 (2.2)	1.9 (2.0)	0.9 (1.3)	0.180	0.446
Figure copying	8.8 (2.4)	10.9 (0.4)	9.0 (2.2)	8.7 (2.7)	0.917	0.791
Boston naming test	9.3 (3.6)	9.9 (3.9)	10.6 (2.6)	10.3 (3.0)	0.284	0.294
Animal naming	9.8 (4.9)	11.8 (5.7)	12.4 (6.1)	10.7 (5.3)	0.649	0.465
Phonemic fluency	31.0 (13.1)	31.7 (11.5)	29.6 (14.5)	28.6 (15.4)	0.746	0.804
Cornell sum	5.6 (4.6)	5.5 (4.7)	6.7 (6.4)	5.0 (4.2)	0.617	0.896
NPI-Q delusions, n/total reg (%)	2/15 (13)	7/26 (27)	3/18 (17)	11/57 (19)	0.593	0.499
NPI-Q hallucinations, n/total reg (%)	0/15	2/26 (8)	5/18 (11)	3/57 (5)	0.364	0.293
NPI-Q sleep, n/total reg (%)	3/15 (20)	13/26 (50)	7/17 (41)	13/57 (23)	0.816	0.311
Cerebral stroke/TIA^b^, n (%)	3 (19)	2 (7)	5 (26)	9 (15)	0.735	0.695
Hypertension, n (%)	5 (31)	13 (45)	7 (37)	28 (48)	0.247	0.305
Diabetes mellitus, n (%)	2 (13)	5 (17)	1 (5)	3 (5)	0.292	0.593
3DT1 sequence, n (%)	8 (50)	9 (31)	13 (68)	33 (24)	0.672	0.917
3.0 Tesla, n (%)	6 (38)	14 (48)	6 (32)	26 (44)	0.638	0.678
CDR-SB baseline	4.3 (1.0)	4.9 (1.8)	4.7 (1.8)	5.4 (1.6)	**0.002**	**0.010**
CDR-memory	0.97 (0.13)	1.07 (0.35)	1.21 (0.51)	1.24 (0.46)	**<0.001**	**<0.001**
CDR-orientation	0.81 (0.31)	0.91 (0.40)	0.89 (0.49)	1.01 (0.43)	0.093	0.184
CDR-judgment	0.84 (0.24)	0.81 (0.36)	0.84 (0.29)	0.91 (0.25)	0.376	0.743
CDR-social	0.63 (0.22)	0.78 (0.37)	0.76 (0.42)	0.91 (0.44)	**0.016**	**0.003**
CDR-hobbies	0.78 (0.26)	1.03 (0.58)	0.89 (0.54)	1.02 (0.45)	0.051	0.092
CDR-personal care	0.31 (0.48)	0.34 (0.48)	0.21 (0.42)	0.34 (0.51)	0.853	0.968
CDR score baseline	0.7 (0.3)	0.8 (0.2)	0.8 (0.2)	0.9 (0.2)	**0.023**	**0.024**
APOE- ɛ4 carrier, %	56.3%	66.7%	72.2%	64.8%	0.533	0.417
Amyloid β, ng/L	681 (290)^1^	438 (193)^2^	534 (143)^3^	502 (158)^4^	**0.028**	**0.007**
Amyloid β, % abnormal^c^	22.2	81.3	40.0	64.0	**0.031**	**0.017**
Total tau, ng/L	768 (437)^1^	701 (383)^2^	891 (319)^3^	615 (267)^4^	0.225	0.566
Total tau, % abnormal^c^	66.7%	62.5%	90.0%	64.0%	0.886	0.907
Phosphorylated tau, ng/L	95 (46)^1^	84 (34)^2^	112 (38)^3^	81 (31)^4^	0.315	0.604
Phosphorylated tau, % abnormal^c^	77.8%	62.5%	80.0%	48.0%	0.123	0.281

All scale measures reported as mean (SD).

*Minimal-atrophy—Typical AD, t-test/ χ^2^-test.

** Minimal-atrophy—All other AD subtypes, t-test/ χ^2^-test.

^a^Age minus symptom duration,

^b^Previous comorbidity/history,

^c^Based on AHUS cut-offs.

^1^ n = 9.

^2^ n = 16.

^3^ n = 10.

^4^ n = 25.

MMSE Mini-mental State Examination, TMT Trail making test, CDR-SB Clinical Dementia Rating scale sum of boxes, NPI-Q Neuro Psychiatric Inventory Questionnaire.

**Fig 1:**
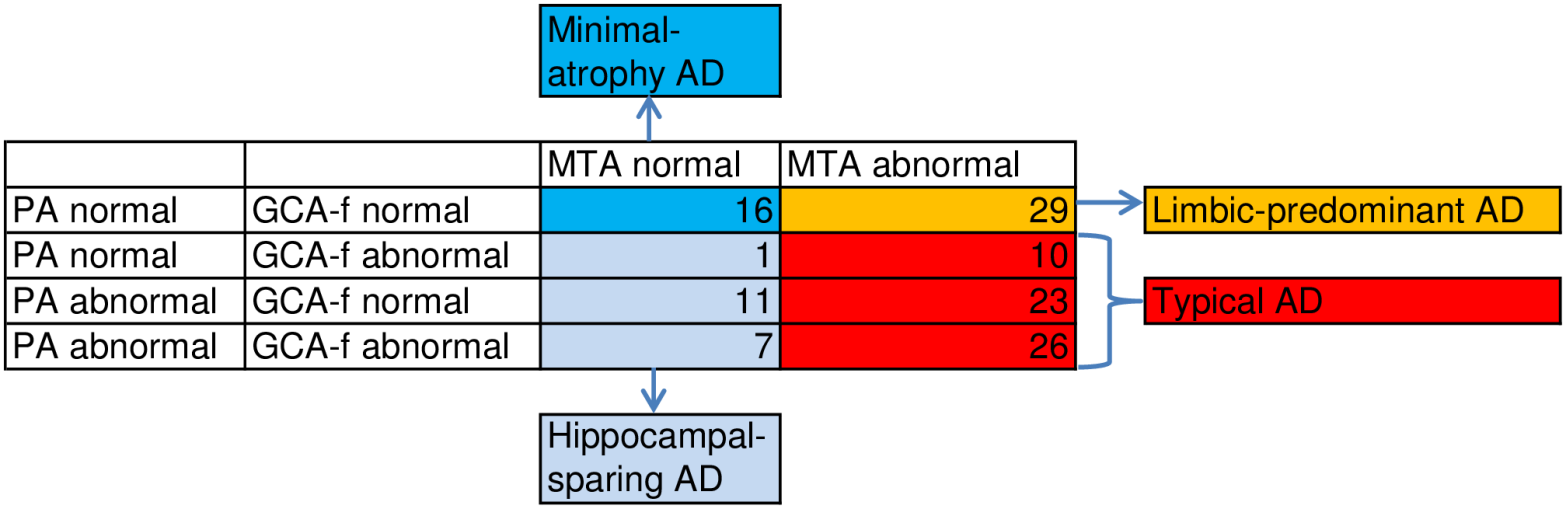
Distribution of AD subtypes. PA = Posterior atrophy ad modum Koedam. GCA-f = Global cortical atrophy-frontal ad modum Pasquier. MTA = Medial temporal lobe atrophy ad modum Scheltens scale.

Recently developed age-adjusted cut-offs [31] were used to evaluate whether atrophy was present in the three separate brain regions and to categorize the patients according to atrophy-pattern subtypes [11]. This subdivision is illustrated in Fig 1. If both the MTA and one or both of the other scales were abnormal, the atrophy pattern was regarded as typical AD. If only the MTA was abnormal, the pattern was regarded as limbic-predominant. If the MTA was normal, and one or both of the other scales were abnormal, the pattern was regarded as hippocampal-sparing. In addition, if all three scales were normal, the pattern was regarded as minimal-atrophy.

### Other assessments

APOE genotyping was conducted using the Illumina Infinium OmniExpress v1.1 chip at deCODE Genetics, Reykjavik, Iceland, and the results were dichotomized based on APOE-ɛ4 status (carrier of at least one APOE-ɛ4 allele, or not).

Lumbar puncture with measures of AD biomarkers (amyloid-β [Aβ], phosphorylated tau [P-tau] and total tau [T-tau]) in the cerebrospinal fluid (CSF) was carried out in 60 of the 123 patients. The CSF examination was done in patients where more information was warranted to increase the etiological diagnostic precision, mostly in younger patients. All CSF samples were analyzed at Akershus University Hospital (AHUS) using ELISA technique with the Innotest kit (Innogenetics, Ghent, Belgium). As the analyses were carried out as part of the clinical routine, the samples were analyzed on different dates and with different batches. The laboratory is part of the Alzheimer’s Association quality-control program for CSF biomarkers through a collaboration with the Clinical Neurochemistry Laboratory in Gothenburg, Sweden [36]. Cut-offs developed at AHUS were used when dichotomizing the results into a pathological/nonpathological variable (normal references: Aβ 550–1200 ng/L, P-tau <80 ng/L, T-tau in patients with ages <50, <300ng/L; in patients 50–70 years, <450 ng/L; in patients with ages >70, <500 ng/L).

### Statistical analysis

The data were analyzed using IBM SPSS Statistics for Windows, version 22.0, Armonk, NY, USA. Demographic and clinical characteristics across subtypes were compared using Student’s t-test and ANOVA for continuous data and with χ^2^-test for categorical data. Logistic regression analyses were performed to further explore differences found in the comparisons between the minimal-atrophy group and typical AD (total n = 74) and between the minimal-atrophy AD group and all other AD subtypes (total n = 122). A p-value below 0.05 was used as a threshold for statistical significance.

### Ethics

The patients and caregivers received oral and written information and gave written consent to participate. Only patients with the capacity to consent were recruited at baseline. Caregivers gave written consent if the patient lacked the capacity to consent at follow-up.

The Regional Committees for Ethics in Medical Research in South-Eastern Norway approved the study (REK 2011/531).

## Results

The mean age of the patients was 74.0 (SD 7.3) years; 56% were females; the mean MMSE was 22.1 (SD 4.3); mean formal education was 11.4 (SD 3.6) years; and the mean annual progression as measured by the CDR-SB change was 2.2 (SD 2.0) for all 123 patients.

**Fig 2:**
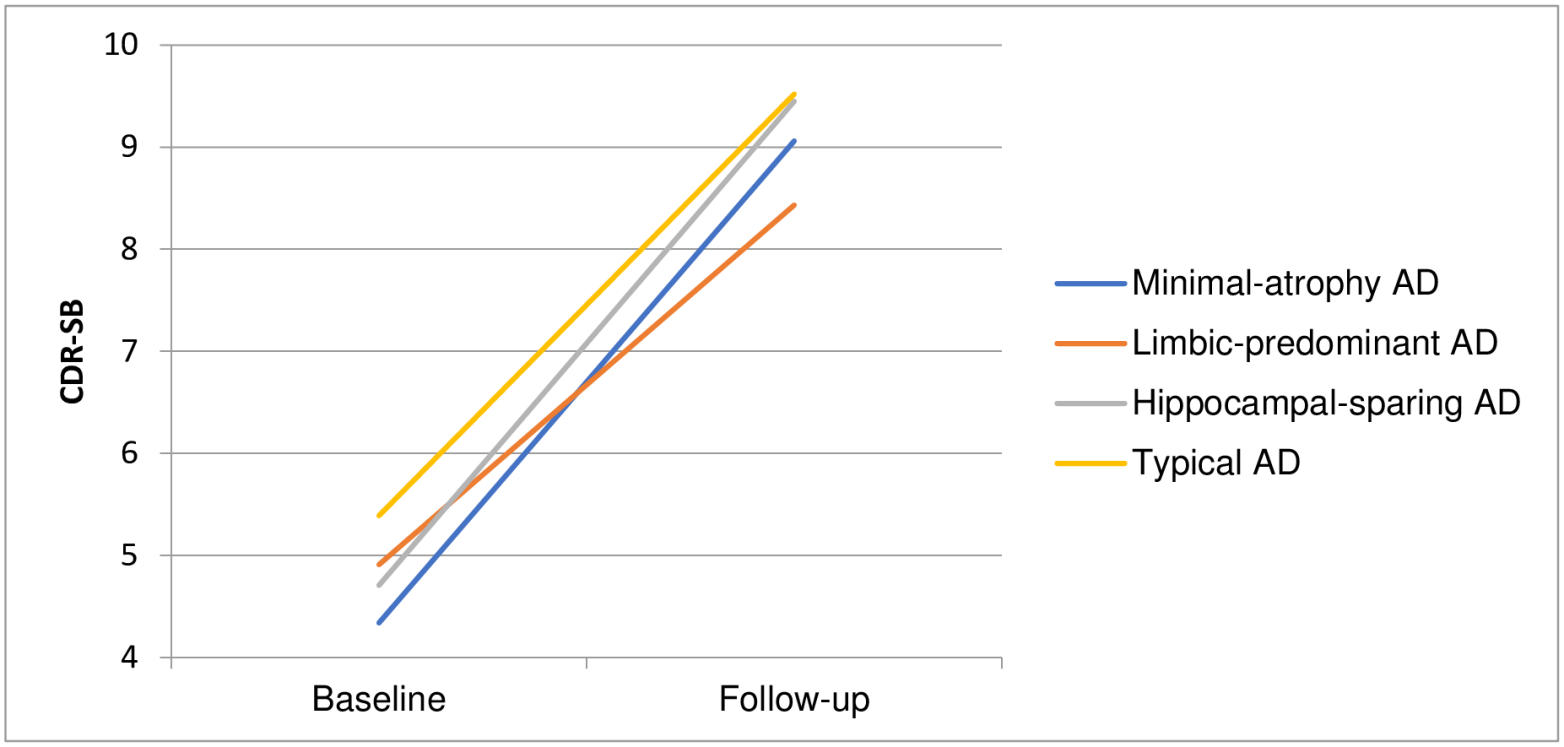
Progression as measured by the CDR-SB at baseline and follow-up.

Table 1 shows the four atrophy-pattern AD subtypes identified through the MRI visual rating scales, with descriptive data: 59 (48%) patients had typical AD; 29 (24%) had limbic-predominant AD; 19 (15%) had hippocampal-sparing AD; and 16 (13%) had the minimal-atrophy AD pattern. No differences regarding demographic, cognitive, APOE-ɛ4, CSF biomarkers, neuropsychiatric symptoms, cerebrovascular comorbidity and risk factors, or progression rate (see also Fig 2) were identified among the four subtypes using ANOVA (not shown). Comparing the minimal-atrophy group with typical AD and all other AD subtypes showed that the number of years of education and CDR-SB score at baseline were lower in the minimal-atrophy group, and the level of Aβ was higher (as was the percentage of patients in the minimal-atrophy group with Aβ levels above the cut-off, i.e., a negative test result). No other differences were found between these groups (Tables 1 and 2).

**Table 2:** Follow-up characteristics of the patients by AD subtype.

	Minimal-atrophy n = 16, 13.0%	Limbic-predominant n = 29, 23.6%	Hippocampal-sparing n = 19, 15.4%	Typical AD n = 59, 48.0%	p*	p**
MMSE follow-up	17.9 (7.1)	20.0 (5.0)	19.0 (4.5)	16.8 (7.0)	0.587	0.965
MMSE annual change	-1.3 (2.4)	-1.6 (2.2)	-1.7 (2.1)	-2.8 (3.0)	0.093	0.209
Delayed recall follow-up	0.7 (1.4)	0.5 (0.8)	1.4 (2.1)	0.3 (0.6)	0.316	0.572
Delayed recall annual change	-0.50 (0.98)	-0.53 (0.84)	0.02 (0.79)	-0.23 (0.55)	0.304	0.249
Phonemic fluency follow-up	22.1 (14.1)	22.9 (10.8)	25.8 (15.5)	17.9 (15.9)	0.371	0.700
Phonemic fluency annual change	-4.6 (6.9)	-5.3 (3.1)	-0.7 (3.7)	-2.7 (6.3)	0.567	0.510
CDR-SB follow-up	9.1 (3.4)	8.4 (4.1)	9.4 (4.4)	9.5 (3.7)	0.662	0.887
CDR-SB annual change	2.5 (1.9)	1.9 (2.2)	2.3 (2.2)	2.1 (2.0)	0.512	0.472
CDR score follow-up	1.5 (0.5)	1.3 (0.7)	1.6 (0.8)	1.6 (0.7)	0.663	0.921
CDR score annual change	0.41 (0.30)	0.29 (0.39)	0.36 (0.38)	0.36 (0.40)	0.662	0.524

All measures reported as mean (SD). “Annual change” calculated as change between follow-up assessment (17–34 months) and baseline assessment, per year.

*Minimal-atrophy—Typical AD, t-test.

** Minimal-atrophy—All other AD subtypes, t-test.

MMSE Mini-mental State Examination, CDR-SB Clinical Dementia Rating scale sum of boxes.

Tables 3 and 4 show the results from the logistic regression analyses comparing the minimal-atrophy group with typical AD and all other AD subtypes, respectively. As can be seen, patients in the minimal-atrophy group had significantly fewer years of formal education and lower CDR-SB at baseline in the adjusted models (models 1, 2 and 3). The score on the MMSE, by contrast, did not differ between the groups, either in unadjusted or adjusted analyses.

**Table 3:** Logistic regression. Minimal-atrophy (0)-Typical AD (1).

n = 74	Unadjusted		Model 1		Model 2		Model 3	
	OR (95% CI)	p	OR (95% CI)	P	OR (95% CI)	p	OR (95% CI)	p
Age	1.02 (0.94; 1.11)	0.570	1.04 (0.95–1.13)	0.401	1.04 (0.95–1.13)	0.425	1.01 (0.92–1.12)	0.766
Female	0.522 (0.16; 1.69)	0.279	0.85 (0.24–3.04)	0.797	0.85 (0.24–3.05)	0.801	1.52 (0.35–6.56)	0.575
Education	1.32 (1.05–1.65)	**0.016**	1.33 (1.05–1.68)	**0.018**	1.31 (1.03–1.68)	**0.028**	1.46 (1.12–1.89)	**0.005**
MMSE	1.10 (0.96; 1.25)	0.175			1.02 (0.89–1.18)	0.769		
CDR-SB	1.76 (1.08; 2.87)	**0.023**					2.17 (1.22–3.87)	**0.009**
Nagelkerke				17.2		17.4		34.3

MMSE Mini-mental State Examination, CDR-SB Clinical Dementia Rating scale sum of boxes. In the unadjusted column, only one variable was included at a time. For model 1, 2 and 3, the variables with presented data were added simultaneously.

**Table 4:** Logistic regression. Minimal-atrophy (0)-All other AD subtypes (1).

n = 122	Unadjusted		Model 1		Model 2		Model 3	
	OR (95% CI)	p	OR (95% CI)	P	OR (95% CI)	p	OR (95% CI)	p
Age	1.01 (0.94; 1.09)	0.708	1.04 (0.96–1.13)	0.367	1.04 (0.96–1.13)	0.380	1.03 (0.94–1.11)	0.565
Female	0.53 (0.17; 1.63)	0.266	0.74 (0.23–2.37)	0.609	0.75 (0.23–2.45)	0.637	0.80 (0.24–2.71)	0.719
Education	1.29 (1.05; 1.60)	**0.017**	1.31 (1.05–1.64)	**0.018**	1.30 (1.03–1.64)	**0.031**	1.43 (1.11–1.84)	**0.005**
MMSE	1.09 (0.97; 1.22)	0.161			1.02 (0.90–1.15)	0.807		
CDR-SB	1.38 (0.97; 1.97)	0.075					1.68 (1.11–2.55)	**0.014**
Nagelkerke				12.8		12.8		22.4

MMSE Mini-mental State Examination, CDR-SB Clinical Dementia Rating scale sum of boxes. In the unadjusted column, only one variable was included at a time. For model 1, 2 and 3, the variables with presented data were added simultaneously.

In total, 60 patients had available CSF biomarkers, of which nine patients belonged to the minimal-atrophy group. When Aβ level was added to model 3 in Table 4 (minimal-atrophy versus all other AD subtypes, n = 59), the Aβ level was, in addition to education and CDR-SB, a significant variable (p 0.038), with higher levels of Aβ in the minimal-atrophy group (data not shown).

## Discussion

Previous neuroimaging studies have identified three subtypes of AD found to correlate well with neuropathological findings of neurofibrillary tangle distribution [6, 9, 12, 37]. In the extension of these findings, a fourth subtype showing no atrophy or minimal atrophy on MRI has recently been recognized [10, 11]. The first aim of the present study was to explore whether the same four subtypes, identified using MRI assessed with three different visual rating scales, would exist in a heterogeneous naturalistic memory-clinic sample, as has been found in two previous studies carried out in the more selective ADNI cohorts [13]. The prevalence of the subtypes identified in the present study was in line with previous findings, with typical AD being the most frequent subtype, occurring in 48% of the patients in the present study compared to 38–59% in previous studies, followed by the limbic-predominant and hippocampal-sparing AD subtypes, occurring in 24% and 15% respectively in the present study compared to 17–29% and 12–18% in previous studies. Finally, the minimal-atrophy subtype represented 13% of the patients, as compared to 10–17% in previous studies [10, 11].

When comparing the minimal-atrophy group to both typical AD and all other AD subtypes, no differences were found with regard to cognitive function. The two previous studies on MRI subtypes of AD found a lower MMSE score in the minimal-atrophy group, but Ferreira et al. concluded that, based on a broader cognitive test battery, there was great overlap between the subtypes [10, 11]. Moreover, in the present study, no significant difference in the progression rate could be found. The only observed differences were that the minimal-atrophy group had significantly fewer years of formal education, a tendency that was also reported in previous studies [10, 11]; in addition, they had a lower CDR-SB score at baseline, and fewer patients had pathological levels of Aβ. These differences remained significant in adjusted analyses.

While the lack of or lower degree of atrophy and higher Aβ level could indicate that the minimal-atrophy AD subtype had been misdiagnosed as AD, we believe that the similar cognitive deficits and progression rate in this group, as compared to the other subtypes, *do* indicate that, clinically, these patients had AD. In addition, they clearly fulfilled the diagnostic criteria for AD [16, 17]. However, there is also a possibility that the minimal-atrophy group could represent other dementia disorders, for instance, dementia with Lewy bodies [38], but as the clinical symptomatology, progression rate, behavioral symptoms like hallucinations and delusions, and degree of cerebrovascular comorbidity or cerebrovascular risk factors were similar compared to the other AD patients, this is unlikely [39].

The question remains unanswered regarding whether the CSF Aβ is false negative, another pathological substrate is actually present in this patient group, or it could be negative because the minimal-atrophy group is at an earlier neuropathological stage.

In summary, we suggest that fewer years of formal education, the lack of MRI-assessed atrophy, and the smaller number of patients with pathological Aβ levels but clinical AD phenotype are findings that could be in line with the cognitive reserve hypothesis (CRH) [40].

The CRH proposes that education is beneficial for the brain, both by producing more efficient and pathology-robust brain networks (neural reserve) and by neural compensation, in which well-developed brain networks can be used in new and unusual ways when the brain is damaged by disease or injury [40]. Furthermore, in patients with similar degrees of cognitive impairment, the underlying pathology of AD has been demonstrated to be more advanced in highly educated patients, while patients with less education show less pathological alteration [40]. We believe that the results of the present study could be in line with this hypothesis. Patients with less education develop symptoms earlier in the neuropathological disease progression when tangles start to form and neurons malfunction and die, even before atrophy can be identified on MRI.

The results of the present study align with other studies that have reported thinner regional cortices or more regional atrophy [41–45] and reduced white matter fiber tract integrity [46] in highly educated AD patients compared to equally impaired patients with less education. Furthermore, similar tendencies related to fewer years of formal education in the minimal-atrophy AD subtype were reported in the studies by Byun et al. and Ferreira et al. [10, 11]. We have found only one study that reported findings in contrast with this. Shpanskaya et al. found a *positive* association between education and hippocampal volume in AD [47]. However, they did not adjust for disease duration or cognitive function. Adding APOE-ɛ4 status to our models, as done by Shpanskaya et al., did not change our results.

As previously noted, we found that the levels of Aβ and the number of patients with negative Aβ results according to cut-offs were higher in the minimal-atrophy group compared to patients of the other subtypes. Bennett et al. concluded that education modifies the relationship of amyloid to cognition, meaning that patients with less education need less amyloid pathology to develop cognitive symptoms [48, 49]. Although our result regarding the minimal-atrophy group having higher Aβ levels could be in line with the CRH, it is in contrast to previous studies reporting equally or even greater pathological levels of Aβ in the minimal-atrophy group [10, 11]. There might be several reasons for this discrepancy. One possible explanation is that, in all the studies, the proportion of patients with CSF biomarkers was between 49% and 67%, which could create selection bias in different ways depending on how patients were selected for CSF analysis.

Concerning the other CSF biomarkers, the minimal-atrophy group had levels of t-tau and p-tau similar to the other groups. Earlier research conducted with mice has found t-tau and p-tau to be secreted from neurons possibly stimulated by Alzheimer-related factors, e.g., amyloid [50]. We further suggest that in humans, these CSF markers therefore do not have to reflect neurodegeneration and tangle pathology directly, and the finding of these patients having high t-tau and p-tau is thus not in contrast to the hypothesis that they have little tangle pathology.

Another discrepancy between the present and two previous longitudinal studies is that, whereas Byun et al. and Ferreira et al. [10, 11] found that the minimal-atrophy group had the slowest progression rate among the groups, we did not find such a difference. In addition, both studies were based on the same selective research cohort (ADNI) and not on a heterogeneous sample of clinical patients, as our memory-clinic cohort was (our cohort possibly including more vascular comorbidity). For these reasons, the studies were not entirely comparable.

One last finding that needs discussion was that the less-educated, minimal-atrophy group had similar test results as the other subtypes regarding the MMSE and other cognitive tests at baseline, while the global score as measured with the CDR-SB (memory and social items more specifically) was lower (indicating better function). This could seem contradictory, but as the CDR should not only reflect cognitive test results but also the functional level as reported by a proxy, we suggest this result indicates that this patient group better maintains well-incorporated overall daily functioning compared to specific cognitive functions. However, as the CDR-SB remains significant after correcting for educational level and substantially increases the explained variance, educational level/CRH does not explain all the difference in CDR-SB between the groups. Possibly, the proxy information is the relevant factor creating this difference, or the atrophy subtype has a more direct impact on cognition and less impact on, or better preservation of, function.

During the last decade, several international working groups have proposed newer ways of diagnosing AD, incorporating knowledge of its neurobiology and the use of biomarkers at different levels of necessity [1, 51]. The finding of less-educated patients with AD lacking cerebral atrophy is important, as these patients, according to several of the suggested criteria, would not receive an AD diagnosis. The results of the present study could also imply that visual rating scales might not be sensitive enough to capture low degrees of atrophy; however, both Ferreira et al. and Byun et al., using automated MRI methods, confirmed the lack of atrophy in the minimal-atrophy group [10, 11].

There are limitations to this study. The risk of the minimal-atrophy group being misdiagnosed as AD has been discussed but is regarded unlikely because their cognitive profiles and progression rates are similar to those of other patients, as well as the fact that they were diagnosed by experienced physicians based on a broad clinical assessment and according to standardized diagnostic criteria. The patients were rediagnosed at follow-up, and all except two retained an AD diagnosis (one with limbic-predominant and one with typical AD pattern). In addition, potential differential diagnoses such as depression and atrophy in other parts of the brain have been regarded in the previous studies. Neither the present study nor the study by Byun et al. found any differences regarding depression [10], and atrophy in other parts of the brain has not been found to differ between the subtypes either [10].

MRI imaging was conducted using different MRI protocols, which might reduce reliability. To reduce variability, only one neuroradiologist performed the visual ratings. The neuroradiologist was used to image heterogeneity through extensive practice in the use of visual rating scales in both clinical routine and research settings [30, 31]. With regard to the T1/T2 contrast and field strength heterogeneity, the distribution was not significantly different among the AD subtypes. While T1/T2 might affect atrophy ratings, a situation that the neuroradiologist is accustomed to and takes into account during ratings, field strength is not found to affect atrophy ratings [52]. Another MRI-related limitation was that the cut-offs used were based on only a single study, however, the cut-offs have been validated in several later studies [53, 54].

The number of patients with available CSF analysis was low, and the risk of selection bias is present. Moreover, CSF samples were not analyzed on the same date with the same batches which might increase variability [55]. Therefore, the CSF results should be interpreted with caution.

Lastly, the number of patients, especially in the minimal-atrophy group, was low. However, similar prevalences as identified in previous studies were found, thereby strengthening the results.

Future studies are needed to test whether the current findings can be replicated. We suggest that adding more measures of cognitive reserve, such as previous and current occupation or measures of intelligence or literacy, would strengthen a future study [56].

## Conclusion

Previous results concerning the prevalence and the similar phenotypic expressions of the four AD subtypes were confirmed. In this clinical sample, no differences in progression rates were found. The main finding was that patients without evident atrophy, as assessed by three visual MRI rating scales, had less education than other patients. We believe this finding may support the cognitive reserve hypothesis. Patients with higher education can cope with more neuropathological changes than less-educated patients can, or put another way, patients with less formal education might present with clinically typical AD symptoms before they have positive biomarkers of AD. This finding represents a challenge that should be considered in the process of developing new diagnostic criteria in order not to lose sensitivity, and it should be kept in mind by physicians examining patients with fewer years of formal education as well as in relation to treatment trials.
